# Cassia Seed Gum Films Incorporated with Partridge Tea Extract as an Edible Antioxidant Food Packaging Film for Preservation of Chicken Jerky

**DOI:** 10.3390/polym16081086

**Published:** 2024-04-12

**Authors:** Na Wei, Zijing Pan, Yuping Ning, Wenhua Liu, Xin Wen, Chen Yang, Lijuan Wang

**Affiliations:** Key Laboratory of Bio-Based Materials Science and Technology of Ministry of Education, Northeast Forestry University, 26th Hexing Road, Xiangfang District, Harbin 150040, China; 2021220612@nefu.edu.cn (N.W.); 15304798909@nefu.edu.cn (Z.P.); ningyp@nefu.edu.cn (Y.N.); 2021121601@nefu.edu.cn (W.L.); 2021210600@nefu.edu.cn (X.W.); yc1357@nefu.edu.cn (C.Y.)

**Keywords:** cassia gum, partridge tea extract, antioxidant activity, packaging film

## Abstract

The use of edible packaging films to delay food spoilage has attracted widespread attention. In this study, partridge tea extract (PTE) was added to cassia gum (CG) to prepare CG/PTE films. The microstructure, optical, mechanical, barrier, and antioxidant properties of CG/PTE films were investigated, and the effect of PTE on CG films was shown. The films had high transparency and smooth surface structure. Additionally, PTE significantly improved the elongation at break and antioxidant activity of films. At 2.5% of PTE, the 2,2-Diphenyl-1-picrylhydrazyl (DPPH) radical scavenging rate of the film was 46.88% after diluting 50 times, indicating excellent antioxidant property, which could be applied to food preservation. After 9 days of storage, the thiobarbituric acid reactive substances values (TBARS) of chicken jerk packaged with films containing 0% and 2.5% PTE increased from 0.12% to 1.04% and 0.11% to 0.40%, respectively. This study suggests that CG/PTE films can be used to preserve cooked meat.

## 1. Introduction

Meat is a popular food due to its high nutritional value. It is in high demand every year and is usually considered the basis of some popular dishes in the world [1]. Because of the plentiful nutrients and high water activity, meat products are easily perishable [2]. If they are not well preserved, the products will spoil and produce many bacteria, which can threaten human health. Currently, lipid oxidation and auto-oxidation are the main reasons for cooked meat deterioration, which can reduce the quality of meat products, cause a shorter shelf life, and decrease nutritional value [3]. According to reports, the oxidation of lipids produces many toxic substances, and the end products of lipid peroxidation are associated with many diseases in the body, such as atherosclerosis, neurodegenerative diseases, and cancer [4]. Furthermore, contaminated ready-to-eat meat products pose a high risk to susceptible populations such as pregnant women, older adults, and those who are immunocompromised. Therefore, it is critical to find the right way to slow down meat spoilage and prolong the shelf life of meat products. Nowadays, the common methods for delaying meat spoilage include refrigeration, ionizing radiation, chemical preservatives, biological preservation, high hydrostatic pressure, and packaging [5]. Among them, the packaging is easy to operate, and it can protect the product from microorganisms, oxidation, contamination, and deterioration. The performance of packaging is often improved by adding some active substances or reagents. For example, deep eutectic solvents (DES) can act as a plasticizer to improve the tensile strength of the film [6]. Poly(vinyl chloride) (PVC) films are prepared by adding a bio-based efficient plasticizer prepared from wasted cooking oil and citric acid [7]. Plastics have the advantages of being high quality, lightweight, easy to process, and low cost, but they are not easy to degrade [8]. When heated at high temperatures, it is likely to release harmful substances into the environment, inducing other issues. In addition, the plastic packaging film can also generate microplastics, which accumulate in the food chain, thus causing harm to the human body and the environment [9]. Furthermore, reports on films based on natural hydrocolloids are numerous, and all kinds of bioactive components have been added to natural hydrocolloids to improve their different properties. Peppermint essential oil and thymol endowed a konjac glucan and carrageenan film with antibacterial and antioxidant activities [10]. The nanocellulose fibers and bioactive compounds from banana peels reinforced a starch-based film’s mechanical, barrier, antimicrobial, and antioxidant performances [11]. Compared to plastic films, natural polymers can be combined with bioactives to produce more environmentally friendly packaging film, which is helpful in preserving foods and alleviating the environmental pollution problem.

Cassia gum (CG) is a hydrophilic colloid derived from the endosperm of the cassia plant; its molecular weight is between 200,000 and 300,000 daltons. CG is a long-chain polymer consisting of 1,4-β-D-mannopyranose units and 1,6-α glycosidic bonds linking galactose in the side chains [12]. It exhibits good biocompatibility, water solubility, and biodegradability due to its large number of hydroxyl groups, and possesses good film-forming ability and can be used as can be used as an antioxidant carrier integrated with natural additives [13]. Nevertheless, cassia gum film suffers from some problems, such as low antioxidant activity and few researches on edible food packaging. In order to improve the properties of CG film, it can be blended with other substances. For example, xanthan gum and cassia gum were mixed to obtain high-viscosity solutions through synergistic interactions [14]. The water resistance, mechanical properties, and barrier properties of CG film have been improved by adding anise essential oil and Pickering emulsion [15]. Furthermore, the pH-sensitive intelligent films based on cassia gum were investigated [16]. However, food packaging films need a certain mechanical strength and barrier properties to protect the food from damage in the process of transportation and storage, as well as some bioactive substances to improve the free radical scavenging rate. Compared to other polysaccharides, cassia gum is less studied in the field of edible packaging. Further improvement is still needed in terms of antioxidant properties.

Partridge tea (*Mallotus furetianus (Bail) Muell Arg.*), also known as hairy tea, is a kind of tropical plant belonging to the family of euphorbiaceae [17]. It is a distinctive local generation of tea beverage plants and medicinal plants in Hainan Province, China. Owing to the presence of phenolic acids, flavonoids, coumarins, polysaccharides and other nutrients [18,19], partridge tea has many biological activities such as being anti-greasy, antioxidant, anti-aging, choleretic, and anti-inflammatory properties [20,21,22]. The partridge tea extracts exhibit high antioxidant activity due to phenols with good solubility in water, therefore, hot water extraction was used in this study. Partridge tea plays a critical role in Hainan’s poverty alleviation, and it is often used in the fields of health food, pharmaceuticals, and cosmetics, but it is rarely used in food packaging. Moreover, there are no reports on the preparation of antioxidant film by adding partridge tea extract as an active substance to the film.

In summary, this study aims to investigate the potential application of a green natural product (PTE) in active packaging. Physicochemical properties of the CG/PTE films were evaluated, including optical properties, mechanical properties, water vapor transmission rate, and antioxidant properties. In addition, scanning electron microscopy (SEM), Fourier-transform infrared (FTIR), and X-ray diffraction (XRD) were used to further investigate the effect of partridge tea extract on CG/PTE films. Furthermore, an edible film containing different concentrations of partridge tea extract was prepared, and its preservation effect on chicken jerky was investigated.

## 2. Materials and Methods

### 2.1. Materials

Cassia gum (food) was supplied by Henan Anli Fine Chemical Co., Ltd. (Zhengzhou, China). Partridge tea was supplied by Hainan Jiufengyan Tea Co., Ltd. (Wanning, China). Glycerol and ethanol were purchased from Tianjin Yongda Chemical Reagent Co., Ltd. (Tianjin, China). The chicken was purchased from a local supermarket (Harbin, China).

### 2.2. Preparation of PTE

The partridge tea leaves powder, with a particle size of 40–60 mesh, was added to 200 mL of distilled water at 80 °C for 2 h. After filtering and cooling, the resulting solution was made up to 500 mL and designated as a PTE.

### 2.3. Preparation of Films

Firstly, 6.0 g of cassia gum was added in 25.0 mL of anhydrous ethanol to favor its uniform dispersion in solution and then mixed with 500 mL of distilled water. After stirring at 60 °C and 300 rpm for 30 min, 2.7 g of glycerol was added to the mixing solution. The amount of PTE added (0%, 0.5%, 1.0%, 1.5%, 2.0%, and 2.5%, based on the quality of CG) was fixed to 100 mL using distilled water and poured into the film-forming solution. After stirring for 30 min, the solution was poured into an acrylic mold (28.0 cm × 29.0 cm × 5.0 cm), left to stand for 10 min, and dried at 60 ℃ for 24 h. The films were named CG/PTE (0.0%), CG/PTE (0.5%), CG/PTE (1.0%), CG/PTE (1.5%), CG/PTE (2.0%) and CG/PTE (2.5%), respectively.

### 2.4. Characterization of Films

#### 2.4.1. Scanning Electron Microscopy (SEM)

The films fractured in liquid nitrogen were gold-sprayed, and the cross sections were observed using a Quanta 200 SEM (FEI Co., Hillsborough, OR, USA) at 5 kV. 

#### 2.4.2. FTIR Analysis

The film samples (1.0 cm × 1.0 cm) were scanned in the range of 4000–500 cm^−1^ using ATR-FTIR with a resolution of 4 cm^−1^.

#### 2.4.3. X-ray Diffraction (XRD)

The film samples (3.0 cm × 3.0 cm) were examined at a rate of 0.16°/s in the range of 5–90° at 40 kV voltage and a test current of 40 mA using an X-ray diffractometer (D/max 2200, Rigaku Corporation, Tokyo, Japan). 

### 2.5. Performance Test

#### 2.5.1. Color of Films

The optical properties of films were investigated using a colorimeter (Datacolor 800, Delta Technologies, Lawrenceville, NJ, USA). The parameters include redness/greenness (a*), yellowness/blueness (b*), and lightness (L*).

#### 2.5.2. Haze

Film haze was tested with a haze meter (CS-700, CHNSpec, Hangzhou, China) by cutting films into 3.0 cm × 3.0 cm square sheets and covering the light outlets. Each group of samples was measured three times, and the average value was determined.

#### 2.5.3. Thickness and Mechanical Properties 

The mechanical properties of films were measured according to the reference [23]. Firstly, films were cut into long strips (8.0 cm × 1.5 cm) and were humidified at 53% RH for 12 h. For the prepared films, 10 measurement points were randomly taken on them, and the thickness was measured using an instrument (D-C11ZXBS, Mitutoyo, Kawasaki, Japan). Then, their tensile strength (TS) and elongation at break (EB) were tested with an intelligent electronic tensile tester (XLW-PC, Languang, Jinan, China) with a tensile speed of 300 mm/min and a load of 500 N. Each sample was measured five times, and the average value was calculated.

#### 2.5.4. Light Transmittance 

The light transmittance of CG/PTE films was measured in the range of 200–800 nm using a UV-visible spectrophotometer (UV-2600, Shimadzu, Kyoto, Japan). The film was cut into squares (2.0 cm × 2.0 cm) and placed in the slot of the UV-visible spectrophotometer, and the measurement mode was set to transmittance.

#### 2.5.5. Water Vapor Permeability (WVP)

WVP was measured via weight change using the moisture-permeable cups to evaluate the film’s moisture barrier capability [24]. A total of 23.0 g of dried CaCl_2_ was placed in weighing bottles as a hygroscopic agent. The film with an area of 18.10 cm^2^ was encapsulated on the mouth of the bottle with a hot melt adhesive. The prepared bottles remained at 0% RH for 12 h to remove the excess water. Then, they were placed in a 75% RH desiccator to absorb humidity. The mass of the weighing bottles was recorded at certain intervals. WVP was calculated using Equation (1):

(1) $$\mathrm{WVP}=\left(\Delta m\times d\right)/\left(S\times \Delta t\times \Delta P\right)$$

where WVP is the water vapor transmission coefficient (g s^−1^ m^−1^ Pa^−1^); ∆m is the mass difference of the vapor permeable cup at different times (g); d is the average thickness of the specimen film (m); ∆t is the test interval time (s); S is the effective area of the specimen (m^2^); ΔP is the difference in vapor pressure between different humidities (Pa); and the difference in vapor pressure is 1753 Pa at 0% RH and 75% RH (25 °C).

#### 2.5.6. Determination of DPPH Free Radical Scavenging Ability

The antioxidant activity of samples was determined according to the method of Yu et al. with appropriate modifications [25]. A total of 4.00 mg of DPPH was accurately weighed and dissolved in 100.0 mL of anhydrous ethanol to obtain the mother liquor. A certain amount of the mother liquor was pipetted and diluted with 50% ethanol until the absorbance at 517 nm was 0.8 ± 0.2 to obtain the working solution of the DPPH radical. Then, 1.50 g of the sample was cut into pieces, and 25 mL of 50% ethanol was added. After shaking at 150 rpm and 25 °C for 12 h, the sample was left to stand for 36 h away from light and diluted 50 times to obtain the solution to be measured. A total of 3.0 mL of diluted solution and 3.0 mL of DPPH working solution were mixed completely in a test tube. After 30 min, the absorbance of the reaction solution was measured at 517 nm. The diluted solution was replaced with 50% ethanol as a blank control. The DPPH-scavenging rate of the samples was calculated according to Equation (2):

(2) $$\mathrm{DPPH}\;\mathrm{scavenging}\;\mathrm{activity}=\left(A_{\mathrm{Control}}-A_{\mathrm{Sample}}\right)/A_{\mathrm{Control}}\;$$

where A_Control_ is the absorbance of the reaction solution of 50% ethanol control; A_Sample_ is the absorbance of the reaction solution of the experimental group.

### 2.6. Packaging Application

A total of 15 g of chicken jerky was packaged in a bag made of CG films containing 0%, 0.5%, 1.5%, and 2.5% PTE. Then, all samples were sealed using a seal tester and stored in a temperature and humidity chamber (LHS-100CH, Yiheng Scientific Instrument Co., Ltd., Shanghai, China) at 4 °C. On days 0, 3, 6, and 9, the packaging of each group of chicken was removed for the relevant tests.

The degree of peroxidation of meat was determined according to the reference [26]. Briefly, 5 g of chicken was homogenized in 7.5% trichloroacetic acid and shaken at 50 °C for 30 min. After filtering, 5 mL filtrate was mixed with 5 mL thiobarbitone (TBA) solution to react at 90 °C for 30 min. The absorbance of the cooled solution was measured using a UV-Vis spectrophotometer at 532 nm.

### 2.7. Statistical Analysis 

SPSS v17.0 (SPSS Inc., Chicago, IL, USA) was used to analyze the data for the Duncan multiple range test with a significant difference level of less than 0.05.

## 3. Results

### 3.1. SEM Observation

**Figure 1:**
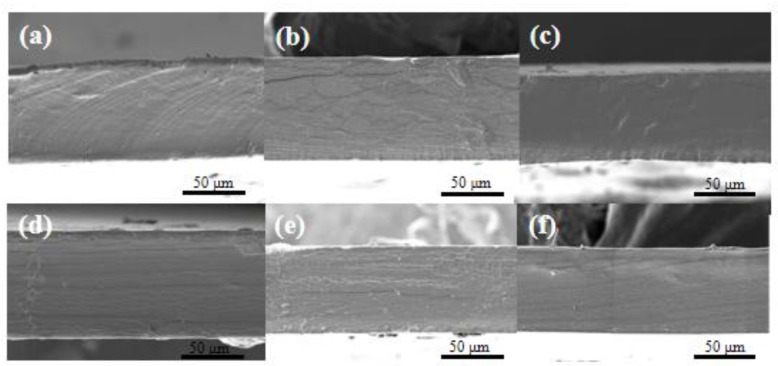
SEM images of the film cross-section with different PTE additions at 50 μm. (**a**) CG/PTE (0.0%); (**b**) CG/PTE (0.5%); (**c**) CG/PTE (1.0%); (**d**) CG/PTE (1.5%); (**e**) CG/PTE (2.0%); (**f**) CG/PTE (2.5%).

The cross-section features of CG/PTE films are illustrated in Figure 1. The cross-section of the CG film had some textures, especially CG/PTE (0.5%) film exhibited cracks, which might be because some H_2_O molecules transformed tiny ice during the quenching process, evaporated, and left cracks in the high vacuum of the gold-spraying process. In fact, with the increase in PTE concentration from 0% to 2.5%, the difference in morphology inside the films was not obvious, and no phase separations were found, indicating that CG and PTE were compatible.

### 3.2. FTIR Analysis

**Figure 2:**
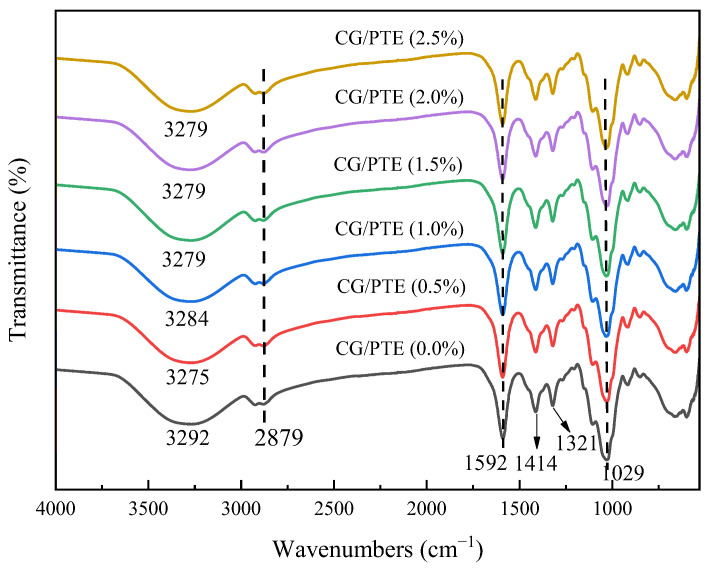
FTIR spectra of films with different PTE additions.

**Figure 3:**
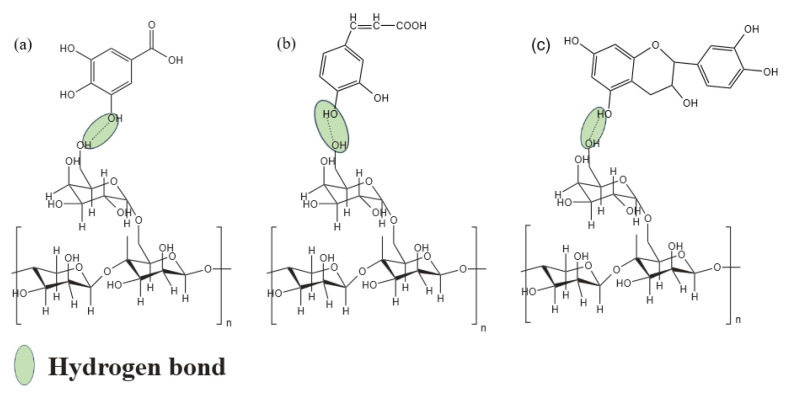
Interaction of cassia gum with the main components in partridge tea extract, (**a**) gallic acid, (**b**) caffeic acid, (**c**) catechin.

The molecular structure and chemical bonding of films can be investigated using IR spectral analysis, and FTIR spectra of the films with different PTE are shown in Figure 2. CG/PTE (0.0%) film showed characteristic bands at 1079 cm^−1^ and 1321 cm^−1^ corresponding to C-O stretching and C-H bending vibrations, respectively. The double peaks at 1591 cm^−1^ and 1414 cm^−1^ were symmetric and asymmetric stretching vibrations of carboxylate [27], which might have originated from glucuronic acid residues on the side chain of the CG molecule. The peak at 2879 cm^−1^ represented the C-H stretching vibrations in methylene and the broad peak at 3292 cm^−1^ was due to the stretching vibrations of conjugated O-H [28]. With the increase in PTE concentration, the position of the characteristic peak of the hydroxyl shifted from 3292 to 3279 cm^−1^, and the intensity of the absorption peak decreased, which indicated that more hydrogen bonds were formed between PTE and CG [13]. The types of hydrogen bonds that might exist between CG and PTE are shown in Figure 3. Compared with CG/PTE (0.0%) film, no new characteristic peaks appeared with the addition of PTE, which suggested that no chemical reaction occurred between PTE and CG.

### 3.3. XRD Analysis

**Figure 4:**
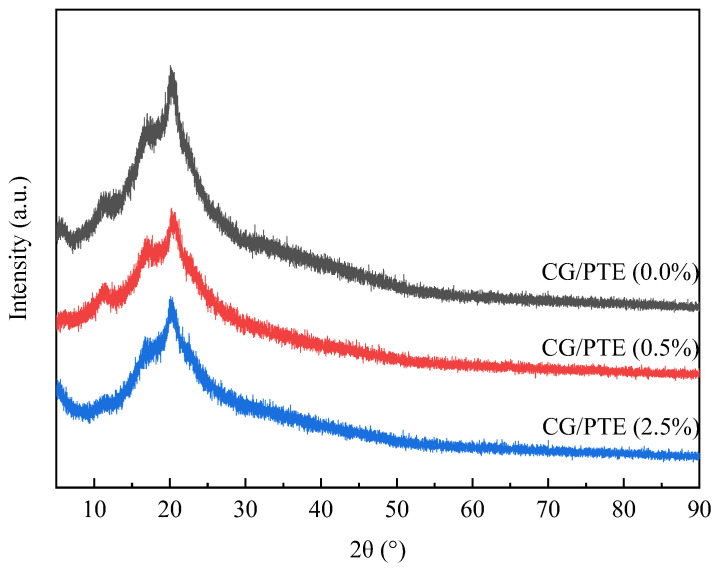
The XRD patterns of CG, CG/PTE (0.5%), and CG/PTE (2.5%) films.

The XRD patterns of CG/PTE (0.0%), CG/PTE (0.5%), and CG/PTE (2.5%) films are displayed in Figure 4. Three diffraction peaks at 11.24°, 16.89°, and 20.27° indicated the presence of crystal structure of CG in the film. The intensity of the peak at 20.27° reduced with the increase in PTE, which meant that the crystallinity of CG/PTE films decreased. This was because the addition of PTE made CG molecules more widely spaced, changing the lattice constant and crystal structure of CG. After adding PTE, the diffraction peak at at 20.27° shifted to 20.45°, and its intensity decreased with the increase in PTE concentration. It might be due to the interaction between PTE and CG, which made the spaces between CG molecules larger, thus reducing the crystallinity of CG film. The diffraction peak of CG/PTE (2.5%) films tended to disappear at 11.24°, indicating the formation of an amorphous structure. It might be attributed to the inhibition of crystal formation by hydrophilic substances with the presence of excess 2.5% PTE, which probably led to a decrease in TS. 

### 3.4. Color Analysis 

**Figure 5:**
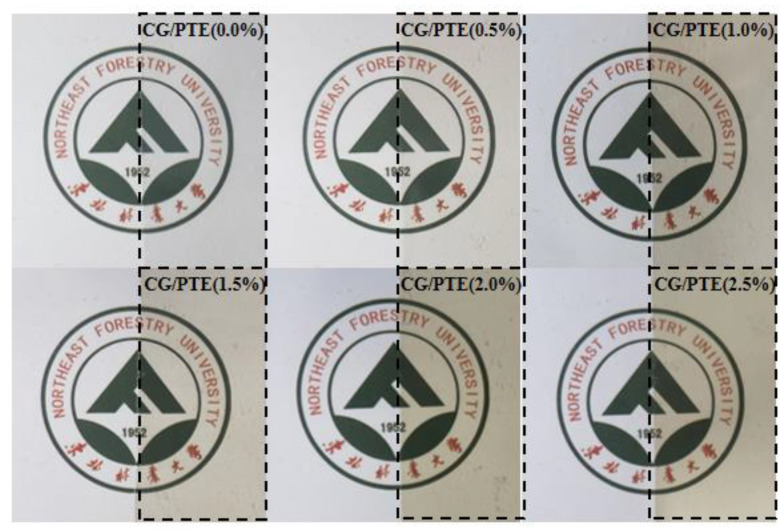
Physical images of the films with different PTE concentrations.

The color parameters of food packaging films determine the acceptability of the film to users. The values of L*, a*, and b* of the films are shown in Table 1, and the physical images of the films with different PTE concentrations are shown in Figure 5. A higher value of L* indicated a higher lightness, the red intensity was expressed as a positive value of a*, while the green intensity was expressed as a negative value of a*. Yellow intensity is expressed as a positive value of b* and blue intensity is expressed as a negative value of b*. Compared to CG film, CG/PTE films possessed lower L* values; moreover, as the concentration of PTE increased, their L* values decreased, indicating that the films became darker. The L* values of CG/PTE (0.0%), CG/PTE (0.5%), CG/PTE (1.0%), CG/PTE (1.5%), CG/PTE (2.0%), and CG/PTE (2.5%) were 91.42 ± 0.03, 89.29 ± 0.03, 88.06 ± 0.02, 83.55 ± 0.13, 82.69 ± 0.3, and 80.54 ± 0.35, respectively. Those might be attributed to the phenolic constituents in PTE extract that caused the scattering and refraction of light, which changed the lightness and darkness of films [29]. Observation of a* and b* values revealed a decrease in the a* value and an increase in the b* value, resulting in a change in film color. It might be due to the yellow color produced by the chromophores of phenol compounds (e.g., C=C and C=O) in PTE [30], which was also visually confirmed. Similar results were also reported when Pickering emulsion was added to CG [15].

### 3.5. Mechanical Properties of CG/PTE Films 

**Figure 6:**
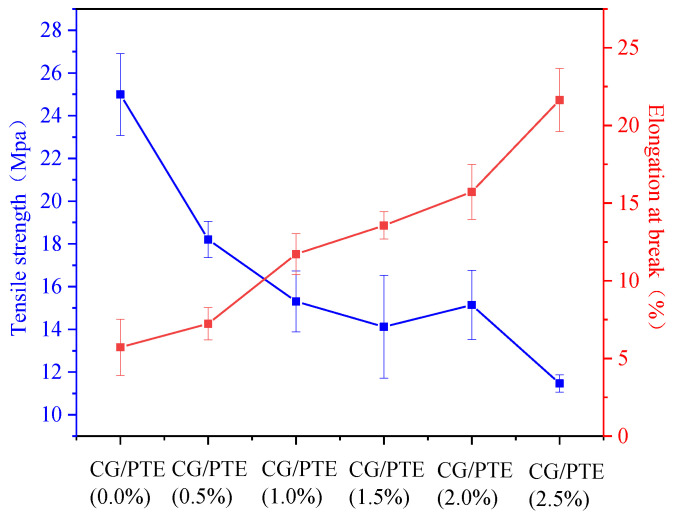
Mechanical properties of films with different PTE additions.

The mechanical properties of CG/PTE films are shown in Figure 6. Tensile strength (TS) and elongation at break (EB) indicated the mechanical resistance, stretching capacity and the flexibility parameters of films, respectively [31]. As the concentration of PTE increased from 0% to 2.5%, TS decreased from 25.0 to 11.5 MPa, and EB increased from 5.72% to 21.64%. Those indicated that PTE acted as a plasticizer, improving the flexibility of films [32]. It might also be due to PTE altering the hydrogen bonding arrangement between glycerol and CG, disrupting the previously packed network structure. Thus, the addition of PTE facilitated the film’s intermolecular movement. The same conclusion could also be demonstrated in the previous XRD analysis. Similar results were observed in the study of pectin, chitosan, and tea polyphenols composite films [33].

### 3.6. Optical Performance 

**Figure 7:**
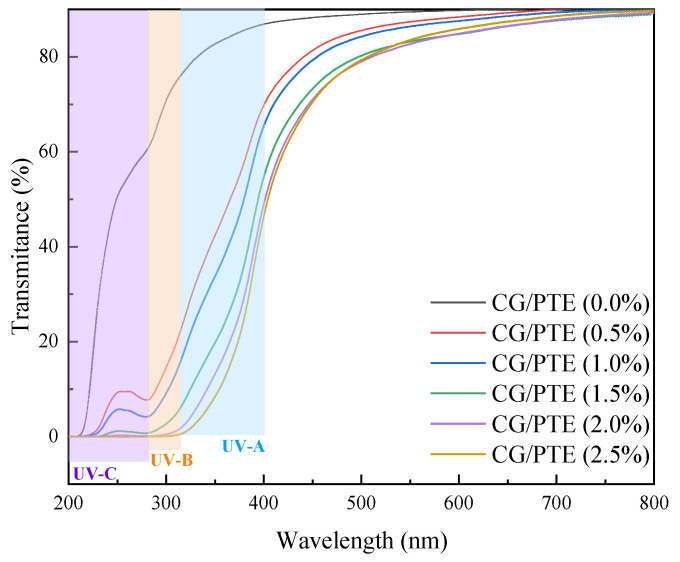
Transmittance of films with different PTE additions.

In transparent food packaging, light reduces food quality and accelerates spoilage [34]. Packaging with low transmittance has a protective effect on the food. The light transmission curves of CG/PTE films are shown in Figure 7, and the haze is displayed in Table 1. As the PTE concentration increased from 0% to 2.5%, the light transmittance of films decreased from 86.69% to 45.88%, and the haze increased from 1.24% to 1.62%, which might be attributed to the fact that PTE increased the disorder of the film structure, leading to enhanced light scattering and reflection. In the wavelength range of 400–760 nm, the transmittance of the films decreased with increasing PTE concentration, and all transmittances were higher than 45%. In the range below 320 nm, the films showed complete shielding against UV light when the amount of PTE was higher than 2%. The patterns packaged by films could be clearly observed, which indicated that the film could satisfy the sensory demand of high visible light transmittance for food packaging in practical application. UV radiation adversely affects food quality through photo-oxidation and photodegradation reactions that produce free radicals and reactive oxygen species, so the UV shielding ability of films is often considered a critical factor in food packaging [35]. Compared to CG film, the transmittance of CG/PTE (2.5%) film was almost 0% in the UVB (275–320 nm) and UVC (200–275 nm) regions. This phenomenon was attributed to the high number of aryl group and phenolic hydroxyl group in the phenols of partridge tea that can effectively absorb ultraviolet light [36], which resulted in the excellent UV adsorption capacity of CG/PTE films; therefore, the addition of PTE reduced the transmittance of the films but improved the UV-blocking ability. Therefore, it could protect food from photo-oxidative deterioration. Yu. et al. found similar conclusions by doping cinnamon essential oil and tea polyphenols to enhance the UV shielding ability of chitosan/polyvinyl alcohol/hydroxypropyl methylcellulose/chymotrypsin composite films [37].

### 3.7. Water Vapor Permeability 

**Figure 8:**
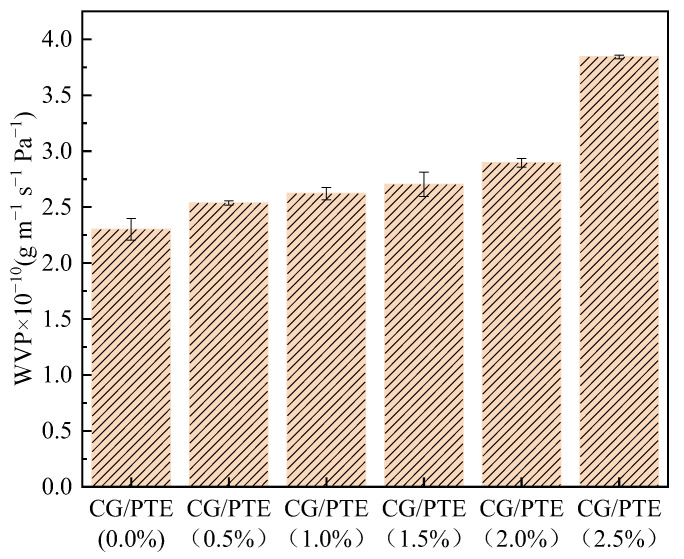
Water vapor permeability of films with different PTE additions.

The WVP of the films is shown in Figure 8. It was found that as the content of PTE increased from 0% to 2.5%, the WVP of films increased from 2.30 × 10^−10^ to 3.84 × 10^−10^ g m^−1^ s^−1^ Pa^−1^, which might be due to the hydrophilicity of PTE. The ability of glycerol to form hydrogen bonds with water molecules also led to high WVP values of films [38]. Additionally, it was possible that the incorporation of PTE reduced the densification of CG film, which increased the diffusion rate of water molecules, leading to an increase in the WVP of the films. The change in molecular spacing was also confirmed using XRD (Figure 4). Han et al. reported that watermelon rind pectin films containing kiwi peel extract possessed a similar increase trend in WVP [39].

### 3.8. Antioxidant Activities of CG/PTE Films 

**Figure 9:**
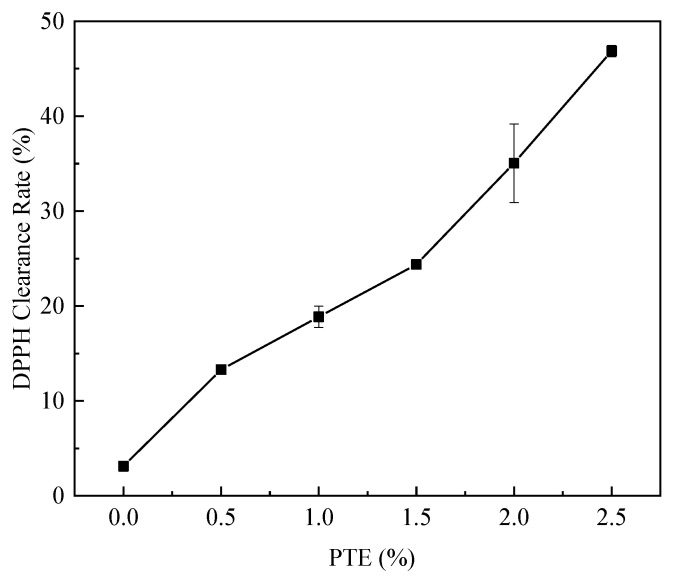
DPPH scavenging rate of CG films with different PTE additions.

Since meat and derived products are easily perishable during improper storage [40], it is crucial to develop a packaging film with a strong antioxidant capacity. DPPH scavenging activity is positively correlated with antioxidant capacity, and the DPPH free radical scavenging test is usually used to determine antioxidant activity. The DPPH radical scavenging rates of CG films containing different PTE concentrations are shown in Figure 9. The DPPH scavenging rate of CG films was 3.13%, indicating that cassia gum itself had a certain antioxidant activity. It could be attributed to the aldehyde groups in the end groups of cassia gum molecules reacted with DPPH radicals As the concentration of PTE increased, the scavenging of DPPH radicals was higher. The scavenging rate was 46.88% when the PTE addition was 2.5%. Phenolic compounds achieved antioxidant activity by providing hydrogen from the hydroxyl group, which acted as an electron donor, eliminating free radicals. Partridge tea phenol has excellent antioxidant and antimicrobial activities. With the increase in PTE concentration, CG/PTE films exhibited excellent antioxidant properties.

### 3.9. Application in Chicken Jerk Preservation 

#### 3.9.1. Appearance 

**Figure 10:**
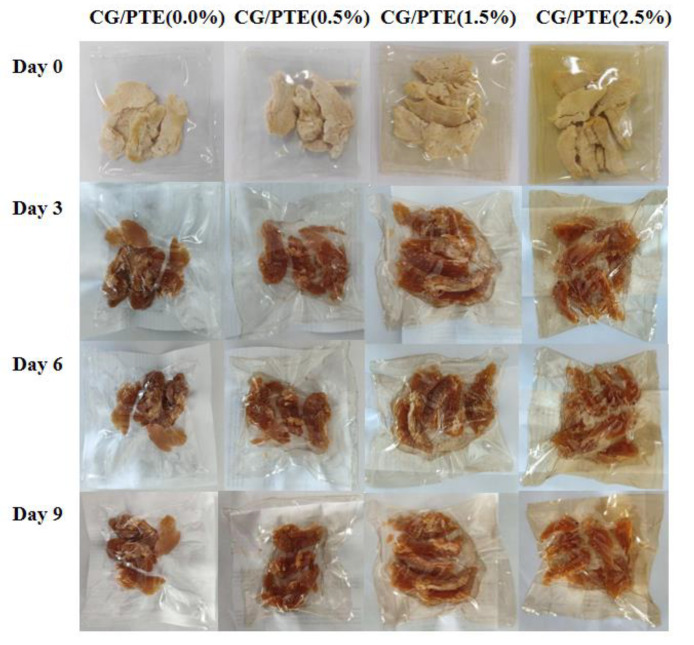
Changes in appearance of chicken jerky packaged with CG/PTE films at days 0, 3, 6, and 9.

We recorded the pictures of CG/PTE films packed on days 0, 3, 6, and 9 and measured the color parameters (L*, a*, and b*) and TBARS as basic indicators for assessing the freshness of chicken jerky. The cooked chicken jerky packaged with CG/PTE films on days 0, 3, 6, and 9 are presented in Figure 10. The color parameters of chicken jerky on day 0 and day 9 are shown in Table 2. With the delay of time, the values of L* decreased, which was consistent with that the surface of the chicken became darked. As the PTE concentration increased from 0% to 2.5%, the L* value increased, but a* values kept decreasing, and b* values decreased slightly and then increased. By comparing each sample, the chicken packaged with CG/PTE (0.0%) became more yellowish-brown. It might result from the characteristic of myoglobin oxidation [41]. The higher L* value of chicken packaged with CG/PTE (2.5%) indicated that the chicken had a paler and fuller appearance. Those could be attributed to the fact that PTE has certain antioxidant properties, which prevented the deterioration of the appearance of the chicken jerky due to oxidation. 

#### 3.9.2. TBARS Analysis 

**Figure 11:**
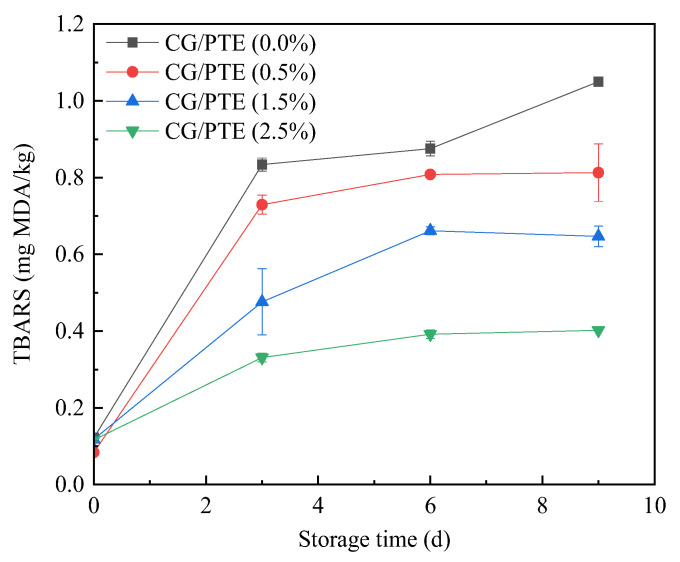
Effect of different storage times on TBARS value of chicken jerky.

TBARS is a reliable indicator to evaluate the degree of lipid oxidation in meat products. As shown in Figure 11, the initial TBARS value of meat samples was 0.1–0.2 mg MDA/kg. As storage time increased, the TBARS values of chicken jerky packaged with CG films added PTE were lower than those of CG film. On day 9, TBARS value of chicken packaged with CG films without partridge tea extract was 1.05 mg/kg. Instead, that with CG/PTE (2.5%) was 0.402 mg/kg. The chicken packaged with CG/PTE (2.5%) film treatment had significantly lower TBARS values than the other groups. Those differences could suggest that CG/PTE films delayed lipid oxidation of meat and prolonged shelf life, which was related to the antioxidant properties of PTE. Similar results were published that chitosan gelatin films incorporating papaya leaf and thyme plant extracts significantly reduced TBARS values of chicken breast fillet during storage [42]. Additionally, a gelatin film of fish skin containing double-layered nanoparticles of tea polyphenol/tricarboxylic acid effectively reduced the TBARS value of air-dried chicken [43]. 

## 4. Conclusions 

In this study, partridge tea extract as an active substance was mixed with cassia gum to prepare CG/PTE films with good antioxidant property. Furthermore, the physical properties of the CG/PTE films were investigated, and their interactions were further analyzed using SEM, FT-IR, and XRD. It was demonstrated that PTE was mixed completely with CG, and the prepared films had a smooth structure. After PTE was added, the films’ flexibility and barrier properties improved, but TS was reduced. The DPPH scavenging rate increased from 3.13% to 46.88% as PTE content rose from 0% to 2.5%, indicating PTE endowed high antioxidant activity to the films. At the end of storage, it made the packaged chicken jerky appear plumper and paler and possessed a lower TBARS value of 0.402 mg/kg compared with CG film. Therefore, the CG/PTE films effectively maintained the quality of the chicken and are promising for packaging oxygen-sensitive foods. 

## Figures and Tables

**Table 1:** Thickness, color parameters, and haze of CG/PTE films.

Film Samples	Thickness (cm)	L*	a*	b*	Haze (%)
CG/PTE (0.0%)	0.127 ± 0.005 a	91.42 ± 0.03 a	2.28 ± 0.04 a	−12.32 ± 0.14 f	1.24 ± 0.01 d
CG/PTE (0.5%)	0.128 ± 0.001 a	89.29 ± 0.03 b	0.45 ± 0.05 b	−4.02 ± 0.09 e	1.32 ± 0.01 c
CG/PTE (1.0%)	0.07 ± 0.002 d	88.06 ± 0.02 c	−0.55 ± 0.02 c	1.44 ± 0.14 d	1.49 ± 0.05 b
CG/PTE (1.5%)	0.084 ± 0.003 c	83.55 ± 0.13 d	−1.64 ± 0.06 d	10.62 ± 0.55 c	1.56 ± 0.03 a
CG/PTE (2.0%)	0.114 ± 0.003 b	82.69 ± 0.30 e	−1.96 ± 0.04 e	14.48 ± 0.73 b	1.62 ± 0.02 a
CG/PTE (2.5%)	0.084 ± 0.001 c	80.54 ± 0.35 f	−1.08 ± 0.15 f	21.07 ± 0.95 a	1.62 ± 0.02 a

Different lowercase letters in the same column indicate significant difference (*p* < 0.05).

**Table 2:** Color parameters of chicken jerky tested on day 0 and day 9.

Color Parameters	Day 0	Day 9			
		CG/PTE (0.0%)	CG/PTE (0.5%)	CG/PTE (1.5%)	CG/PTE (2.5%)
L*	76.58 ± 0.29 a	22.76 ± 0.09 d	32.51 ± 0.52 c	48.60 ± 0.30 b	48.94 ± 0.21 b
a*	1.20 ± 0.10 c	9.49 ± 0.03 a	6.52 ± 0.57 b	6.90 ± 0.06 b	6.18 ± 0.36 b
b*	17.16 ± 1.40 c	28.49 ± 3.58 a	23.53 ± 1.44 b	28.26 ± 0.38 a	31.94 ± 0.53 a

Different lowercase letters in the same row indicate significant difference (*p* < 0.05).

## Data Availability

Data are contained within the article.
